# Reproducibility and accuracy of optic nerve sheath diameter assessment using ultrasound compared to magnetic resonance imaging

**DOI:** 10.1186/1471-2377-13-187

**Published:** 2013-12-01

**Authors:** Jochen Bäuerle, Florian Schuchardt, Laure Schroeder, Karl Egger, Matthias Weigel, Andreas Harloff

**Affiliations:** 1 Department of Neurology, University Medical Center Freiburg, Breisacher Str. 64, 79106 Freiburg, Germany; 2 Department of Neuroradiology, University Medical Center Freiburg, Freiburg, Germany; 3 Department of Radiology, Medical Physics, University Medical Center Freiburg, Freiburg, Germany

**Keywords:** Optic nerve sheath diameter, Transbulbar sonography, MRI, HASTE, TSE

## Abstract

**Background:**

Quantification of the optic nerve sheath diameter (ONSD) by transbulbar sonography is a promising non-invasive technique for the detection of altered intracranial pressure. In order to establish this method as follow-up tool in diseases with intracranial hyper- or hypotension scan-rescan reproducibility and accuracy need to be systematically investigated.

**Methods:**

The right ONSD of 15 healthy volunteers (mean age 24.5 ± 0.8 years) were measured by both transbulbar sonography (9 – 3 MHz) and 3 Tesla MRI (half-Fourier acquisition single-shot turbo spin-echo sequences, HASTE) 3 and 5 mm behind papilla. All volunteers underwent repeated ultrasound and MRI examinations in order to assess scan-rescan reproducibility and accuracy. Moreover, inter- and intra-observer variabilities were calculated for both techniques.

**Results:**

Scan-rescan reproducibility was robust for ONSD quantification by sonography and MRI at both depths (r > 0.75, p ≤ 0.001, mean differences < 2%). Comparing ultrasound- and MRI-derived ONSD values, we found acceptable agreement between both methods for measurements at a depth of 3 mm (r = 0.72, p = 0.002, mean difference < 5%). Further analyses revealed good inter- and intra-observer reliability for sonographic measurements 3 mm behind the papilla and for MRI at 3 and 5 mm (r > 0.82, p < 0.001, mean differences < 5%).

**Conclusions:**

Sonographic ONSD quantification 3 mm behind the papilla can be performed with good reproducibility, measurement accuracy and observer agreement. Thus, our findings emphasize the feasibility of this technique as a non-invasive bedside tool for longitudinal ONSD measurements.

## Background

In recent years, assessing the optic nerve sheath diameter (ONSD) by transbulbar sonography and MRI has become a useful tool for the non-invasive detection of altered intracranial pressure. Several studies showed a close association between ONSD enlargement and raised intracranial pressure in patients with severe head injury, intracranial bleeding or idiopathic intracranial hypertension [1-3]. Consistently, a decreased ONSD was found in patients with intracranial hypotension [4]. Furthermore, Dubost et al. found an increase of the ONSD in intracranial hypotension after performing an epidural blood patch [5]. The close correlation of raised intracranial pressure with enlarged ONSD can be explained by the continuity of the meninges and the subarachnoid space into the orbita surrounding the optic nerve [6,7].

Previous studies demonstrated high intra- and interobserver reliability of ONSD quantification using transbulbar ultrasound [8,9]. However, data regarding scan-rescan reproducibility of this technique are missing but are prerequisite before B-mode sonography can be applied for the monitoring of ONSD changes over time. Furthermore, only limited data exist on the evaluation of measurement accuracy of ultrasound [10,11]. MRI of the optic nerve sheath seems to be an ideal reference due to the high spatial resolution and the clear delineation of orbital structures. However, the limited availability and the high costs of MRI restrict its wider use as follow-up tool.

Therefore, our aim was to systematically determine the scan-rescan reproducibility and observer variabilities of ultrasound-based ONSD measurements. Since previous studies on the accuracy of transbulbar sonography have reported conflicting results [10,11] we compared sonography with MRI of the optic nerve sheath. In addition, we investigated the influence of different measurement depths (3 and 5 mm behind the papilla) on the accuracy of ONSD quantification.

## Methods

### Study population

Between July and August 2012, 15 healthy volunteers were prospectively included in the study. Written informed consent was obtained from all persons before entering the study. All subjects were aged 18 years or older and had no history of neurological disorders, amblyopia or diseases of the optic nerve. The study was approved by the local ethics committee of the Albert Ludwigs University Freiburg, Germany, and was performed in accordance with the ethical standards laid down in the 1964 Declaration of Helsinki.

Transbulbar sonography and MRI were carried out on the same day and both were repeated during a second visit 28 ± 11 (range 9 – 53) days later. For each volunteer, only the right eye was evaluated because two measurements on a paired organ are not independent and may bias the results.

### Transorbital sonography

**Figure 1:**
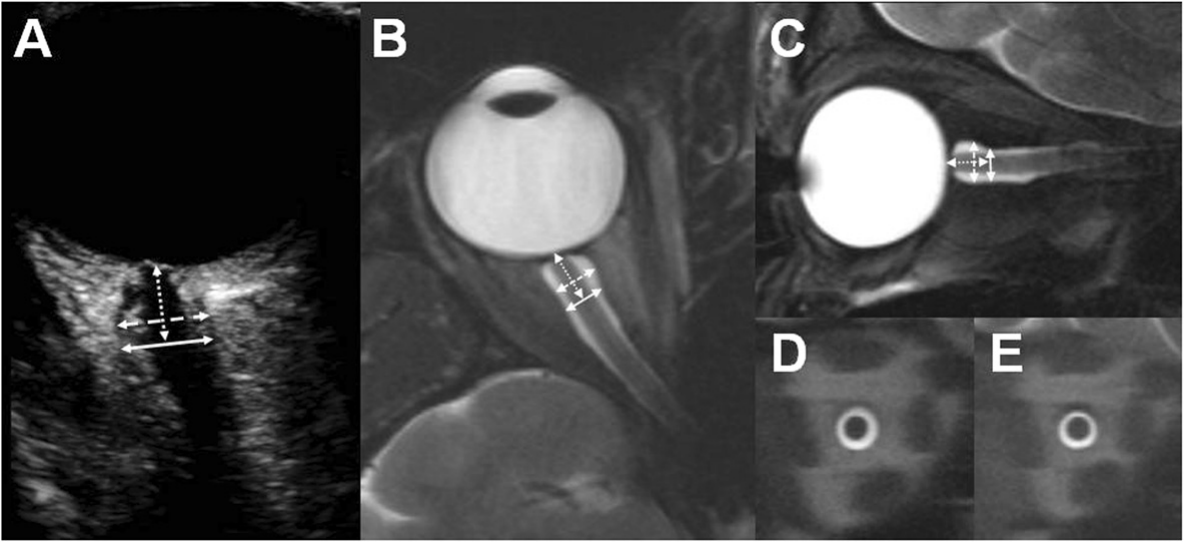
**Transbulbar sonography and MRI of the optic nerve sheath. (A)** The eye bulb and the optic nerve are displayed by B-mode sonography. The optic nerve sheath diameter was measured 3 and 5 mm behind the papilla (dotted arrow) in an axial plane showing the optic nerve in its longitudinal course. Transverse **(B)** and sagital **(C)** T2-weighted TSE sequences were created to schedule coronal and orthogonal HASTE sequences of the optic nerve sheath 3 mm **(D)** and 5 mm **(E)** behind the papilla.

Ultrasound examinations of the ONSD were carried out in B-mode using a Philips iU22 ultrasound system and a 9 – 3 MHz linear array transducer (Philips Medical Systems; Bothell, WA). After a resting time of five minutes volunteers were examined in supine position with the upper part of the body and the head elevated to 20-30°. For safety reasons of biomechanical side effects the mechanical index (MI) was reduced to 0.2, the thermal index (TI) to 0.0. The ultrasound probe was placed on the temporal part of the closed upper eyelid using a thick layer of ultrasound gel. The anterior part of the optic nerve was depicted in a transversal plane showing the papilla and the optic nerve in its longitudinal course. ONSD was assessed 3 mm and 5 mm behind the papilla, as described previously [9,12,13]. In order to gauge the ONSD, the distance between the external borders of the hyperechoic area surrounding the optic nerve was quantified. Each optic nerve sheath was examined three times and means were calculated and considered for further evaluation (Figure 1).

Measurements were carried out by two experienced sonographers (JB and AH). They performed investigations of both eyes of all study subjects independently and were blinded to the results of each other. In order to determine scan-rescan reproducibility observer 1 examined all subjects at both visits. For calculation of inter-observer variability, observer 2 quantified ONSD in two volunteers at the first visit and in 8 volunteers at the second visit. Five individuals were measured on a third visit by both sonographers. To evaluate the intra-observer variability observer 1 did repeated measurements offline using sonographic images that were recorded at the first visit after 75 ± 8 days (range 60 – 86).

### MR imaging

MRI was performed on a 3 T whole-body scanner (Magnetom TIM Trio, Siemens Healthcare, Erlangen, Germany) using a 32-channel phased-array head coil. The employed MRI sequence protocols for ONSD measurements were based on the methodological setup described by Weigel et al. [14]. Prior to the volunteer study MRI protocols were optimized for a 32-channel head coil regarding resolution and contrast versus signal-to-noise-ratio. Subjects were instructed to fixate on a target inside the scanner, with the right eye in straight gaze.

Two different variants of a T_2_-weighted turbo spin echo (TSE) sequence were employed [10,14,15]: (1) A fast T_2_-weighted overview TSE which provides good soft tissue contrast and morphological data for planning: TR = 4000 ms, TE = 130 ms, echo train length = 25, bandwidth = 120 Hz/pixel, ‘weak’ chemical fat saturation. The sequence was applied twice with nine contiguous slices in sagittal (FOV = 21 x 21 cm^2^, Matrix = 448 x 448) and transversal (FOV = 21 x 18 cm^2^, Matrix = 448 x 392) orientation leading to a nominal spatial resolution of 0.47 x 0.47 mm^2^ with slice thickness = 3 mm. The acquisition time was 1:06 min (transversal) and 1:10 min (sagittal), respectively (Figure 1). (2) A rapid T_2_-weighted half-Fourier acquisition single-shot turbo spin-echo (HASTE) sequence that was primarily optimized for quantification: TR = 1700 ms, TE = 129 ms, number of excitations = 1, bandwidth = 196 Hz/pixel, FOV = 19 x 16 cm^2^, Matrix = 448 x 378, phase encoding direction left to right, nominal spatial resolution = 0.42 x 0.42 mm^2^, slice thickness = 2 mm. Acquisition time was 1.7 s per slice. Two slices were acquired perpendicular to the optic nerve orientation within the intraorbital track, guided by the morphological TSE images: the first slice 3 mm behind the papilla, the second slice in a depth of 5 mm (Figure 1).

The ONSD was measured on coronary section HASTE images by drawing spherical regions of interest (ROIs) around the external border of the cerebro spinal fluid (Figure 1). ROI evaluations were performed by a radiologist (KE) and a physician with a one-year experience in neuroradiology (FS) using a state-of-the-art radiology workstation (IMPAX EE R20 VIII P1, Agfa HealthCare N.V., Mortsel, Belgium). For improved visualization, HASTE images were magnified 7.5-fold. As shown by Weigel et al. [14], the accuracy of measurement in the chosen sequence and protocol setup is limited by the reproducibility of ROIs rather than the nominal resolution. This is possible since the experimental setup employs the partial volume effect together with the a-priori knowledge of very high signal cerebro spinal fluid adjacent to low signal parenchymal structures.

Images created during the first visit were assessed by both readers independently. These scans were reanalysed by observer 1 in order to determine intra-observer agreement. For evaluation of the scan-rescan reproducibility observer 2 quantified the ONSD at the second visit.

### Statistics

Values were expressed as mean ± standard deviation. Reproducibility, intra- and inter-observer variability as well as method comparisons were analyzed using the approach by Bland-Altman by calculating the mean difference (d) and standard deviation of the difference. From these data, the limits of agreement were calculated (σd, 95% confidence intervals). Additionally, intra- and inter-observer variabilities were evaluated using the Kappa coefficient. Furthermore, correlation analyses were performed with Pearson’s correlation coefficient (r) to quantify the strength of agreement.

## Results

### Baseline characteristics

ONSD was measured by both ultrasound and MRI in the right eyes of 15 healthy volunteers (mean age 24.5 ± 0.8 years). Ten participants were female. The mean body-mass index was 21.8 ± 3.1 kg/m^2^.

### Optic nerve sheath diameter measurements

**Figure 2:**
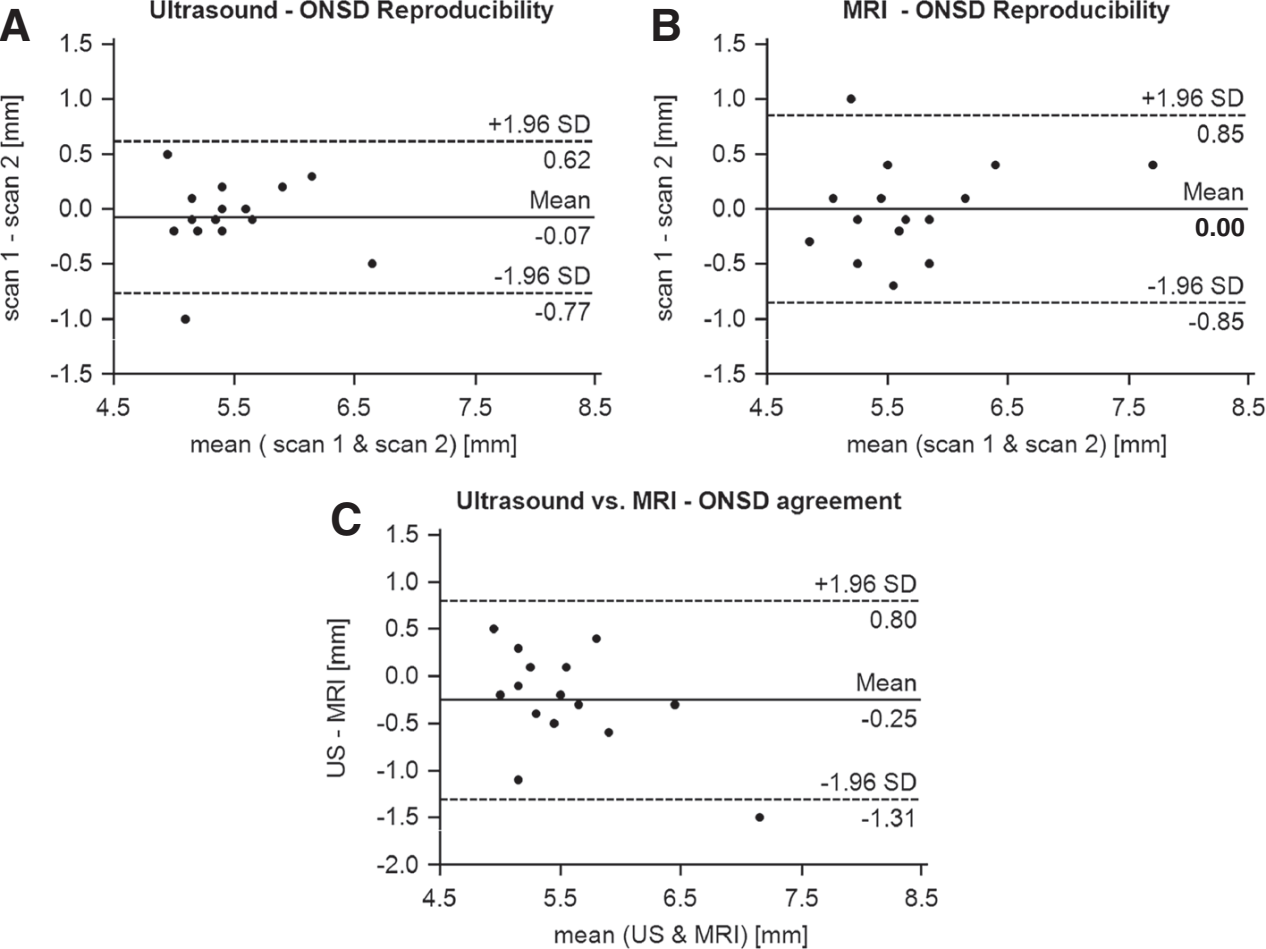
**Reproducibility and accuracy of ONSD assessment.** Bland-Altman plots displaying the agreement of scan-rescan measurements of the optic nerve sheath diameter (ONSD) 3 mm behind the papilla by transorbital sonography **(A)** and MRI **(B)**. Panel **C** demonstrates the agreement between sonographic and MRI-based ONSD quantification at the first visit. Continuous lines depict the mean of differences; dashed lines denote limits of agreement (mean ± 1.96 times of standard deviation).

As summarized in Table 1, ONSD quantification by transbulbar sonography and MRI 3 mm behind the papilla demonstrated good reproducibility (r > 0.75, p < 0.001) with mean differences of < 2% of average ONSD values (Figure 2). If measurements were performed 5 mm behind the papilla Bland-Altman analyses revealed similar results.

**Table 1:** Bland-Altman and correlation analysis of ultrasound and MRI results

	**Descriptive statistics**	**Reproducibility**				**Inter-observer**				**Intra-observer**			
		**(scan 1 vs. scan 2)**				**variability**				**variability**			
		**Bland-Altman**		**Correlation**		**Bland-Altman**		**Correlation**		**Bland-Altman**		**Correlation**	
	**Mean ± SD (range)**	**d**	**σd**	**r**	**P value**	**d**	**σd**	**r**	**P value**	**d**	**σd**	**r**	**P value**
**Ultrasound**													
ONSD 3 mm	5.43 ± 0.49	−0.07	±0.69	0.75	0.001	0.25	±0.51	0.82	<0.001	−0.01	±0.22	0.97	<0.001
	(4.6 – 6.4)												
ONSD 5 mm	5.53 ± 0.57	−0.11	±0.66	0.82	<0.001	0.52	±1.12	0.62	0.01	−0.09	±0.47	0.90	<0.001
	(4.8 – 6.8)												
**MR imaging**													
ONSD 3 mm	5.69 ± 0.77	0.00	±0.85	0.83	<0.001	−0.27	±0.36	0.98	<0.001	−0.01	±0.26	0.99	<0.001
	(4.7 – 7.9)												
ONSD 5 mm	5.09 ± 0.66	−0.01	±0.87	0.76	0.001	−0.14	±0.39	0.97	<0.001	0.01	±0.25	0.98	<0.001
	(4.2 – 7.1)												

d, mean difference; σd, limits of agreement; r, correlation coefficient.

Assessment of the inter-observer variability showed mean differences < 5% of average ONSD values between observers for measurements by both methods 3 mm behind the papilla. Additionally, correlation coefficients (r ≥ 0.82; p < 0.001) indicated a robust inter-observer agreement. Compared to MRI, ONSD quantification by ultrasound at a depth of 5 mm demonstrated an increased inter-observer variability (r = 0.62, p = 0.01, mean differences 9.4%). Intra-observer agreement of ONSD measurements by transbulbar sonography and by MRI at both depths was excellent (r > 0.90, p < 0.001, mean differences < 2%).

These results were confirmed by analyzing the Kappa coefficient. With regard to the inter-observer variability it indicated a moderate strength of agreement for sonography at depth of 3 mm (K = 0.43) and a moderate to good agreement for MRI at both measurement points (3 mm: K = 0.56, 5 mm K = 0.64). 5 mm behind the papilla sonographic measurements matched only fairly (K = 0.27). Additionally, Kappa coefficient showed good intra-observer variability using ultrasound (3 mm: K = 0.83, 5 mm K = 0.63) and MRI (3 mm: K = 0.83, 5 mm K = 0.80) in both depths.

Figure 2 and Table 2 illustrates the direct comparison of transbulbar sonography and MRI. ONSD examinations performed 3 mm behind the papilla by ultrasound revealed a mean difference of < 5% of average ONSD values. Furthermore, correlation showed a close relation between both methods (r = 0.72; p = 0.002). Results for the depth of 5 mm yielded a similar correlation (r = 78; p < 0.001) but a mean difference of 8.6% of average ONSD values.

**Table 2:** Comparison of transorbital sonography and MR imaging

	**Scan 1**			
	**Bland-Altman**		**Correlation**	
	**d**	**σd**	**r**	**P value**
ONSD 3 mm	−0.25	±1.06	0.72	0.002
ONSD 5 mm	0.43	±0.82	0.78	<0.001

d, mean difference; σd, limits of agreement; r, correlation coefficient.

## Discussion

The major finding of our study is the good scan-rescan reproducibility and the robust observer-agreement of both ultrasound and MRI for ONSD assessment 3 mm behind the papilla. To our knowledge this is the first study investigating the scan-rescan reproducibility of sonographic ONSD quantification. Lagrèze et al. already reported good reproducibility for the MRI-based approach [10]. With regard to the scan-rescan reproducibility the wider limits of agreement may reflect small intra-individual fluctuations of the ONSD over the time which may be explained by different physical conditions at the beginning of the examination, for example. In order to reduce such possible effects we examined the volunteers after a short rest.

Slightly differing mean ONSD measurements between observers were evident with regard to sonographic and MRI examinations indicating a systematic bias. However, we believe that these observer dependent variations of < 5% are acceptable for clinical applications. ONSD measurements at a depth of 5 mm were less reliable in ultrasound but highly accurate in MRI. This can be explained by the clear delineation of the ONSD in MRI regardless of depth. In contrast, increased blurring of the optic nerve sheath occurs in B-mode ultrasound at more central parts of the optic nerve (Figure 1). Another factor contributing to this discrepancy may be the unfavourable insonation angle of transorbital sonography and its limited horizontal resolution [10].

The second important aspect of our study was the direct comparison of ONSD measurements between transbulbar sonography and MRI. Since MRI exhibits a higher spatial resolution and a more representative calculation of the mean diameter due to the derivation from the ROI of the optic nerve sheath area it was defined as reference in our study. Recently, Steinborn et al. reported a good correlation between ultrasound and MRI of the ONSD 3 mm behind the papilla in children [11]. Similar results were documented in a cadaver study [16]. Otherwise, Lagrèze et al. found no such relationship in healthy adults with measurements at a depth of 5 mm [10]. Our analysis revealed a mean difference of < 5% ONSD values at 3 mm done by ultrasound and MRI (Figure 2). In addition, limits of agreement were in line with results published by Steinborn et al. [11]. Otherwise, MRI exhibits wider limits of agreement which may be due to the wider range of ONSD values found by MRI compared to sonography. Furthermore, correlations confirmed a close relationship between both methods and highlight the accuracy of transbulbar sonography. In contrast, ONSD at 5 mm seems to be overestimated by sonography, whereas MRI measurements reflect the physiological narrowing of the optic nerve sheath in its intraorbital course [6]. This could be explained by the above mentioned limitations of the sonographic approach. Additionally, the differing measurement planes used by sonography (axial) and MRI (coronary) may account to these findings. Our results suggest that sonographic ONSD quantification should be performed in a depth of 3 mm behind the papilla as recommended by Helmke et al. previously [12,13].

Our study in 15 volunteers with a mean age of ~25 years was not primarily designed to establish representative normal values of the ONSD. However, ONSD values at 3 mm matched closely with those described in previous investigations. Weigel et al. yielded a mean ONSD of the anterior part of the optic nerve of 5.7 mm in healthy adults using the same MRI system and similar sequence protocols [14]. Additionally, Bäuerle et al. proposed a mean ONSD of 5.4 mm for the sonographic approach in previous studies [1,9]. In accordance with these studies we have observed a wide range of ONSD values in healthy adults by sonography and MRI (4.6 – 6.4 vs. 4.7 – 7.9). This finding represents a major limiting factor in predicting altered intracranial pressure by ONSD quantification and suggests that ONSD values should only be interpreted in conjunction with clinical data and neuroimaging results. Discrepancies to studies which indicated a smaller ONSD by sonographic measurements [7,10] may be explained by examiner experience, different interpretation of the imaging anatomy and the use of different ultrasound systems [11].

Our study is limited by the fact that intracranial pressure was presumed to be normal by taking history. Otherwise intracranial masses were ruled out by MRI. Furthermore, the results of this investigation can only be applied on healthy adults. In patients with intracranial hyper- or hypotension sonographic ONSD depiction may be altered leading to deferring test variabilities. This should be the focus of further investigations. Additionally, the results may be limited by the small number of study participants. Nevertheless, we found similar test variabilities than Steinborn et al. [11] documented in 65 children.

## Conclusion

Sonographic ONSD assessment 3 mm behind the papilla can be performed with robust scan-rescan reproducibility and good inter- and intra-observer agreement. Furthermore, compared to MRI transorbital B-mode sonography exhibits reliable measurement accuracy. Accordingly, this technique seems to be suitable for longitudinal investigations of the ONSD. Cost-effectiveness and permanent availability make the sonographic approach highly beneficial. Otherwise, with regard to short acquisition times HASTE sequences of the optic nerve sheath should be implemented in brain MRI examinations in order to receive an easy and comprehensive marker of intracranial pressure.

## Abbreviations

ONSD: Optic nerve sheath diameter; HASTE: Half-Fourier acquisition single-shot turbo spin-echo sequences.

## Competing interests

The authors have no competing interests to declare.

## Authors’ contributions

All authors were substantially involved in conceptual design of the study. JB, FS and AH contributed to the statistical analysis of the data and drafted the manuscript. JB and AH carried out the sonographic examinations. FS and KE did the magnetic resonance imaging assessment. LS substantially contributed to the acquisition of volunteers and coordinated sonographic examinations and magnetic resonance imaging. Further, MW and KE gave substantial technical input regarding magnetic resonance imaging. LS, KE, and MW critically reviewed the manuscript and gave important intellectual input. All authors have critically reviewed and approved the final version of the manuscript.

## Pre-publication history

The pre-publication history for this paper can be accessed here:

http://www.biomedcentral.com/1471-2377/13/187/prepub
